# Identification of electromyographic patterns of bradykinesia in patients with Parkinson's disease

**DOI:** 10.1016/j.heliyon.2024.e39014

**Published:** 2024-10-05

**Authors:** Nikita Kozulin, Anastasiya Migulina, Denis Mokrushin, Gurgen Soghoyan, Aleksandr Artemenko, Artur Biktimirov

**Affiliations:** a Laboratory of Experimental and Translational Medicine, School of Medicine and Life Sciences, Far Eastern Federal University, Vladivostok, Russia; b Vladimir Zelman Center for Neurobiology and Brain Rehabilitation, Skolkovo Institute of Science and Technology, Moscow, Russia; c Department of Neurosurgery, Medical Center, Far Eastern Federal University, Vladivostok, Russia

**Keywords:** Parkinson's disease, Electromyography, Bradykinesia, Levodopa, MDS-UPDRS

## Abstract

**Background:**

Parkinson's disease (PD) is a common neurodegenerative disease characterized by rest tremor, rigidity, and bradykinesia. Assessing the severity of these symptoms is typically done using the third part of the Movement Disorder Society-Sponsored Revision of the Unified Parkinson's Disease Rating Scale (MDS-UPDRS III), relying on subjective evaluations by neurologists, which may lead to challenges in result interpretation. To address this issue, incorporation of surface electromyography (sEMG) in diagnostics.

**Objectives:**

The aim of the study is to search for specific sEMG patterns that allow assessing the severity of bradykinesia.

**Method:**

This case-control study involved 8 patients with PD at Hoehn & Yahr stages 2–3, and 7 healthy volunteers. sEMG was measured while the subjects performed the "finger tapping" and "hand movements" tests of the MDS-UPDRS III. The tests were conducted both before and after levodopa intake to identify patterns indicating changes in motor functions. During the tests, we observed the peak activity of the sEMG signal, reflecting the moments of activation of individual muscle groups involved in the implementation of the movement. Peak activity was characterized by the total number of maximum sEMG signal extrema and the distance between them. The data were compared with the healthy group.

**Results:**

Peak activity increased after levodopa intake, indicating a reduction in bradykinesia. This feature partially correlates with clinicians' assessments and enhances the similarity of predictions by the MDS-UPDRS III scoring model to physician scores.

**Conclusions:**

The results show the effectiveness of using sEMG and the number of peaks corresponding to the moments of muscle activation to assess bradykinesia.

## Highlights

• To objectively identify bradykinesia, we developed a feature called “number of peaks”.
• The number of peaks in sEMG signals indicates a change in velocity of hand movements.
• The number of peaks after L-DOPA intake nears the indicators of the healthy group.
• The model trained on the extended set demonstrated high reliability in predictions.
• Results demonstrate the feasibility of using sEMG in the assessment of bradykinesia.

## 1 Introduction

Parkinson's disease (PD) is one of the most prevalent neurodegenerative disorders, frequently observed in the elderly [1]. To date, there is no cure to halt the neuronal degeneration in PD, which is the cause of the motor symptoms characteristic of this disease. Neuropathological features include the loss of dopaminergic neurons in the substantia nigra, the presence of Lewy bodies resulting from the aggregation of α-synuclein, and neuroinflammation in the brain [2]. This leads to a decrease in dopamine levels in the basal ganglia, resulting in the symptoms of PD [3].

Several internal and external risk factors for PD have been identified, which may affect disease risk differently in men and women [4]. External factors include lifestyle, physical factors (e.g., trauma), and environmental agents (e.g., toxic substances and infectious organisms) [4]. Internal factors consist of genetic predisposition, comorbid conditions (e.g., diabetes), and metabolic status (e.g., elevated urate levels) [4]. Notably, constipation (a well-known prodromal feature of Parkinson's disease associated with Lewy body pathology) and physical activity are highlighted as significant risk factors, providing сlass 1 evidence for their association with PD risk [5].

All therapeutic modalities are directed solely towards mitigating the symptomatic manifestations. One of the most common treatments for PD is dopamine replacement therapy. However, the use of exogenous dopamine is limited because it is a water-soluble, hydrophilic molecule that cannot cross the blood-brain barrier [6]. Levodopa, the direct precursor of dopamine, is the gold standard for PD therapy, as it can cross the central nervous system, where it undergoes decarboxylation to form dopamine [7]. Consequently, levodopa replenishes dopamine levels in the basal ganglia and restores signaling through dopamine receptors, accounting for its therapeutic effect [3].

Classical motor symptoms of PD include: bradykinesia, rigidity, resting tremor, and postural instability, which manifests as unsteadiness during walking and standing [8]. Rigidity is characterized by increased resistance during passive movements of major joints, due to heightened tone in the antagonistic muscles. Tremor interruption results in cog-wheel phenomenon rigidity, where movement becomes intermittent [9]. According to contemporary definitions, tremor is an involuntary, rhythmic, oscillatory movement of a body part [10]. Specifically, resting tremor refers to a tremor with a frequency of 4–6 Hz that occurs when the body part is at rest, often while the limb is supported on an object (e.g., when the hand rests on the armrest of a chair) [9]. Nevertheless, most patients have non-motor manifestations, which often dominate in the clinical presentation [9], thereby complicating the diagnosis of PD, especially in its early stages. Common non-motor symptoms of PD include: sleep dysfunction, autonomic dysfunction (constipation, daytime urinary urgency, symptomatic orthostasis), hyposmia, and psychological disorders (depression, anxiety, or hallucinations) [9].

According to Movement Disorder Society PD (MDS-PD) criteria, the centrality of the motor syndrome remains a key characteristic by which the clinical form of PD is determined [9]. The MDS proposes a two-step process of PD diagnosis [9]. The first step involves the identification of parkinsonism defined as bradykinesia, in combination with rigidity, rest tremor, or both [9]. In the second step, following the identification of parkinsonism, absence of absolute exclusion criteria are identified to specifically attribute the condition to PD [9]. This step also includes supportive criteria (positive features that increase confidence of the PD diagnosis), and "red flags", which necessitate counterbalance through supportive criteria for the definitive diagnosis of PD [9].

Despite the foundational significance of bradykinesia in PD diagnosis, challenges exist in precisely defining this term. The modern definition of "bradykinesia" combines the concepts of bradykinesia (slowness) and akinesia/hypokinesia (decreased amplitude of movement) [9]. Both phenomena are typically identified during examination, although not always concurrently (i.e., patients may be unable to move at a normal speed and amplitude simultaneously) [9]. The current definition of bradykinesia is not necessarily applicable to all cases of parkinsonism. Clinical experience indicates that the signs of bradykinesia may vary among patients, even in those with a confirmed diagnosis of PD [11]. Moreover, isolated bradykinesia may be observed in individuals with non-Parkinsonian disorders (essential tremor, dystonia). For this reason, a proposal has been advanced to reassess the approach to defining bradykinesia [11]. Nowadays, bradykinesia is defined as a reduction in the velocity of executing specifically voluntary movements, whether singular or repetitive [11]. The clinical presentation, incorporating a confluence of bradykinesia with the sequence effect and any additional features, characterizes parkinsonism [12]. Isolated bradykinesia lacks specificity for this particular pathological state [11].

While the criteria proposed by MDS-PD are innovative and complete, they still lack pathological validation [12]. The severity of PD symptoms is commonly evaluated using the Movement Disorder Society-Sponsored Revision of the Unified Parkinson's Disease Rating Scale (MDS-UPDRS). The MDS-UPDRS consists of 50 questions that cover various aspects of PD [13]. It includes four specific sections: non-motor and motor experiences of daily living (Parts I and II, respectively), motor examination (Part III), and motor complications (Part IV) [13]. However, challenges exist regarding the interpretability of this scale. Specifically, the third part of the MDS-UPDRS (MDS-UPDRS III), which assesses a patient's motor functions, relies entirely on the subjective judgment of the clinician. Due to variability among observers and the inability to estimate certain movement parameters accurately with the naked eye, diagnostic inaccuracies may occur [[14], [15], [16]]. Consequently, it becomes imperative to employ additional techniques to enhance the objectivity of the assessment.

It is possible to utilize surface electromyography (sEMG) as an additional diagnostic criterion. The efficacy of computed metrics in interpreting this method for recording physiological activity has been previously acknowledge. sEMG is used to detect rest tremor and postural tremor in patients with PD[17,18]. Various metrics that characterize a signal in time, frequency, and frequency-time domains. Different sets of features are computed to allow training of classifiers. These include sets of features introduced by Du [19] and Hudgins' [20]. Particularly, Hudgins' set comprises the Mean Absolute Value (MAV), Zero Crossings (ZC), Waveform Length (WL) and Slope Sign Change (SSC). Meanwhile, Du's set consists of the integrated absolute value (IAV) of the electromyogram, sEMG variance (VAR), WL, ZC, SSC and Willison Amplitude (WAMP) [21].

Despite the abundance of different metrics, there is still no consensus regarding the preferred use of a specific features. Due to its characteristics, sEMG is infrequently used in the assessment of active fine motor skills. Therefore, we assume the efficacy of sEMG in evaluating MDS-UPDRS III tests is rather limited, as more attention is dedicated to tremor detection.

The aim of the study is to search for specific sEMG patterns that allow assessing the severity of bradykinesia. In this study, we measure and analyze the sEMG signals recorded during patients' performance of the MDS-UPDRS III tests 3.4 "finger tapping", where the patient taps their index finger against their thumb as quickly and with as large a movement as possible, and 3.5 "hand movements", where the patient opens their hand fully and as quickly as possible, both before and after taking levodopa.

## 2 Material and methods

### 2.1 Participants

**Fig. 1:**
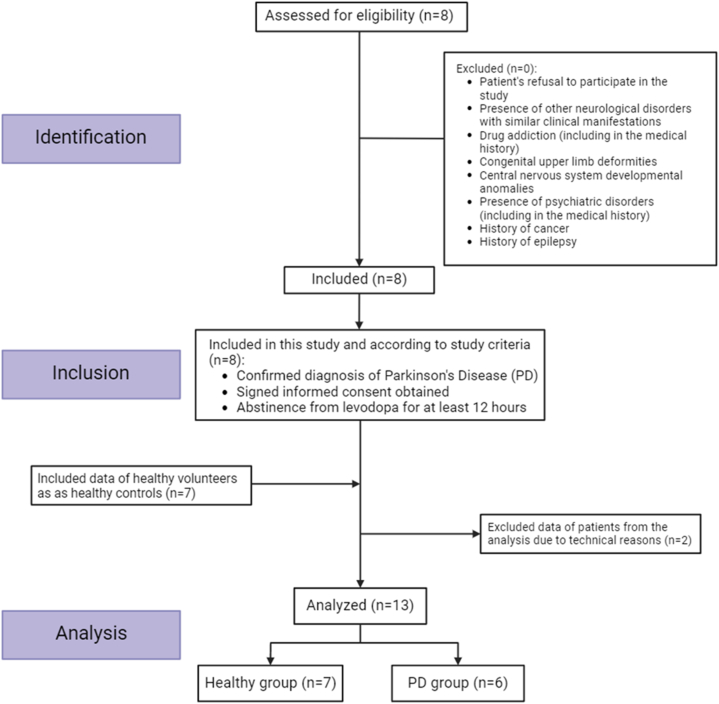
STROBE flow diagram.

Eight patients receiving outpatient treatment at the Medical Center of the Far Eastern Federal University (Medical Center of FEFU) were recruited for the observational, non-randomized case-control study. sEMG data recording for the patients with PD was conducted over two days: one part of the patients was recorded on October 16, 2022, and the other part on October 21, 2022. The process of selecting and including participants illustrated in Fig. 1.

The diagnosis of PD was established according to the MDS-PD criteria [9]. Clinical assessment of PD was conducted using the Hoehn and Yahr scale and the MDS-UPDRS [9]. Each study participant provided voluntary written informed consent, signed after explaining the potential risks and benefits and the nature of the upcoming study. The study protocol was approved by the Committee on Biomedical Ethics (Review board decision December 17th, 2020, protocol No.2) and carried out in accordance with the Declaration of Helsinki. The patients were selected according to the inclusion and exclusion criteria (Supplementary material # Inclusion and exclusion criteria). Age and characteristics of the disease course are detailed in Table 1.

**Table 1:** Demographic and clinical characteristics.

Age (in years)	64,2 ± 7,8
L-DOPA equivalent daily dose (in mg)	583,3 ± 292,3
Hoehn & Yahr stage	2,6 ± 0,4
Disease duration (in years)	11 ± 7,4
*MDS-UPDRS III OFF*	38,5 ± 16,6
*MDS-UPDRS III ON*	25 ± 13,8

Patients underwent an MDS-UPDRS III motor impairment assessment before taking L-DOPA (OFF condition) and after (ON condition). The ON and OFF conditions reflect the motor fluctuations that occur in patients with PD during treatment with levodopa. The ON period begins after the patient takes levodopa and continues as long as the drug acts optimally. During the OFF period, the effects of medication wears off, leading to the return of parkinsonian features [22,23]. The MDS-UPDRS III scores for the entire part and for each item were given by a neurologist-parkinsonologist.

Within the study, a comparison group comprising 7 volunteers was included as healthy controls. They underwent the same tests from the MDS-UPDRS III scale as the PD group. sEMG data recording for the healthy control group took place in the summer of 2022 at the Medical Center of FEFU.

### 2.2 Design of experiment

Patients underwent testing twice on the same day under both ON and OFF conditions. To ensure a confirmed OFF condition, representing the typical motor function in the absence of levodopa, subjects abstained from medication for 12 or more hours [24]. Subsequently, sEMG was recorded during the performance of the MDS-UPDRS III "finger tapping" and "hand movements" tests. Following this, patients ingested a dose of levodopa exceeding the daily prescribed amount by 1.5 times. Upon achieving a full ON condition (30–60 min post-drug intake), a second registration in the ON condition was conducted while adhering to the aforementioned conditions. Simultaneously, the classical assessment of the MDS-UPDRS III test was carried out in parallel with the patient's registration. The entire procedure was documented through video recording (Supplementary material #Video Finger tapping).

Supplementary data related to this article can be found online at https://doi.org/10.1016/j.heliyon.2024.e39014

The following are the Supplementary data related to this article.

Video 1 Video 1

**Fig. 2:**
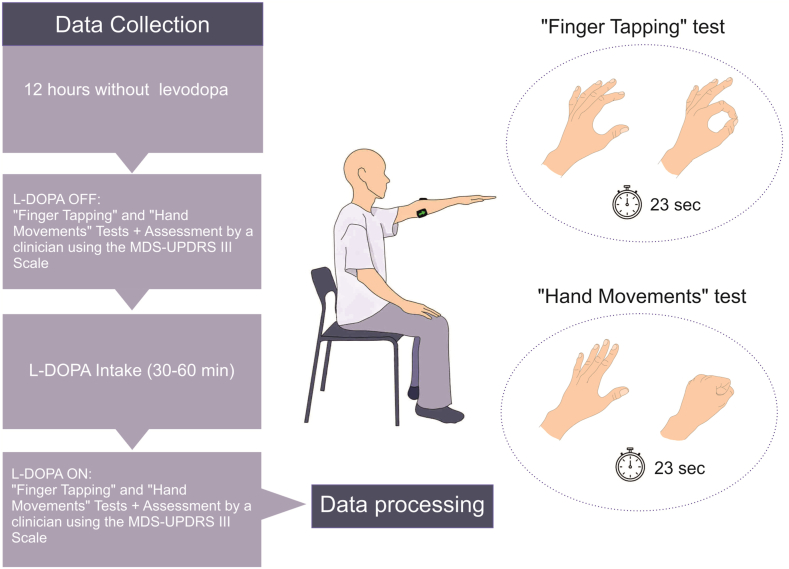
Design of experiment. On the left is the data collection algorithm, detailing the conditions and tests. On the right is the ''Finger Tapping'' and ''Hand Movements'' tests in progress. In the center, the patient's position during the experiment is shown.

During registration, the patient was seated on a chair opposite the operator at several meters. Before starting each test, the operator gave detailed instructions and demonstrated correct performance. The test began and ended at the operator's verbal command and lasted 23 s (Fig. 2).

In the "finger tapping" test, the patient was required to tap his index finger on the thumb as quickly as possible and with the maximum possible amplitude of motion. To perform the "hand movements" test, the patient was instructed to clench the hand into a fist and unclench it as quickly and completely as possible. In this case, the subject's forearm was bent in such a way that the palm was facing the operator.

### 2.3 Recordings

**Fig. 3:**
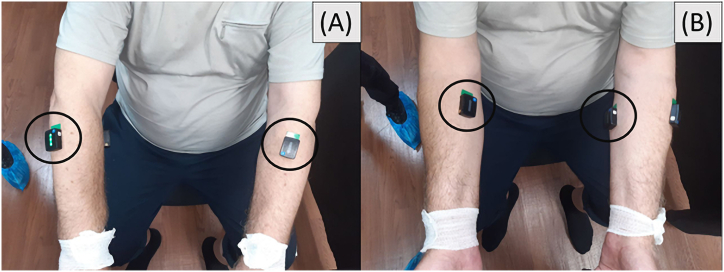
The localization of the sensors. A) Flexor muscle group. B) Extensor muscle group.

The sEMG data was recorded using the Delsys Trigno Wireless Biofeedback System equipped with wireless sensors. The sensor body size is 27x37 × 13 mm, there are two recording electrodes, featuring two recording electrodes spaced 10 mm apart. The data were recorded with a sampling rate of 1260 Hz and a bandwidth of 20–450 Hz. Sensors were placed on the forearm, on the flexor and extensor muscle groups. The localization of the corresponding muscles was determined by palpation of the forearm during wrist movements; the position of the sEMG sensors is illustrated in Fig. 3(A and B). The sensor placement procedure adhered to SENIAM recommendations: the skin areas of the subjects were preliminarily cleaned of hair with a disposable medical razor and degreased with isopropanol 1 min before placement [25].

### 2.4 Data analysis

**Fig. 4:**
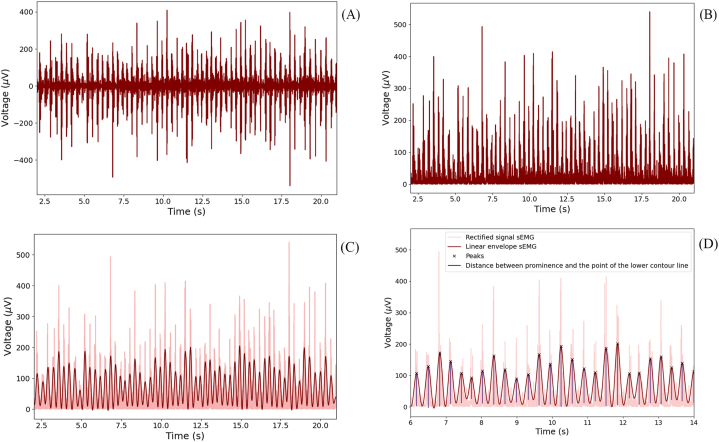
Finding the peaks. А) The processed sEMG signal. B) Rectified signal sEMG. C) Linear envelope of signal. D) Finding prominence by the distance between the highest point of the peak and the point of the lower contour line.

The obtained sEMG data were processed and analyzed using *Python* and "*pyemgpipeline*" library [26]. Data from two patients were excluded from the analysis due to technical reasons. Signal preprocessing involved the removal of baseline DC-offset and subsequent 8th-order Butterworth bandpass filtering with a bandwidth of 10–100 Hz (Fig. 4 A). To eliminate possible artifacts related to the initiation and termination of the subjects' movement, for the first and last 2 s cut off. Subsequently, rectification was performed (Fig. 4 B), and a linear envelope was constructed (Fig. 4C). Based on the constructed envelope, following features were developed to analyze the received signals.

− Number of peaks (maximums) corresponding to the moments of muscle activation;
− Median amplitude, characterizing the power of contractions;
− Average time interval between found peaks.

In order for each considered peak in the signal to be independent and correspond to a specific moment of muscle activation, its prominence was determined (Fig. 4 D). The calculation was based on the vertical distance between the highest point and the point that is the lower contour line, which made it possible to isolate the peak from the signal. As a normalization in each sEMG signal, the minimum prominence distance was calculated, which served as a threshold for identifying the others.

Median of peaks magnitudes characterized the median power of muscle contractions throughout the test. While the distance between the found peaks along the X axis characterized the interval between each period of muscle activation.

### 2.5 Validation of machine learning methods to automate assessments

Previously, quantitative characteristics of the sEMG signal were used to predict the assessment of a neurologist in motor tests [21]. Diverse metrics were used as an input for machine learning models that predicted MDS-UPDRS score that was manually assessed by clinicians during a "finger tapping" test. Here we reproduce the described method on the "finger tapping" and "hand movements'' tests in order to evaluate the effectiveness of the proposed architecture for our data. Next, we integrated additional metrics into the learning process - two modifications of the mean absolute value (MAV1 and MAV2), approximate entropy (ApEn) and sample entropy (SampEn) from Ref. [18], as well as the features we developed – number of peaks and the average time interval between them. Extraction of sEMG characteristics was carried out in the time domain using the *NeuroKit2* [27] and *PySioligy* [28] libraries. The processed signal was segmented into 1 s fragments with an overlap of 500 ms using the Hanning window. Characteristics were determined from each window and then averaged for each sEMG channel. For all the metrics standardization was carried out.

In accordance with the original study [21], a random forest regressor from the Scikit-learn package was used to predict clinical scores. The model was trained using leave - one - subject out cross-validation with 6-folds. In each fold data from one patient from both conditions ON and OFF was assigned to the testing dataset, the rest patients were used to train the models. The predicted MDS-UPDRS III values from all the folds were correlated with real expert estimates. The results from two models were compared. The "Standard model" was developed using the set of metrics from the original study [21]. The "Extended model" integrates features from the standard model with the above described features of the MAV1 and MAV2, ApEn, SampEn, number of peaks, and the average time.

### 2.6 Statistical analysis

Statistical processing of the obtained characteristics of the sEMG signal was carried out using MS Excel 2019 package and Python. For the statistical analysis of changes in sEMG features in the healthy and PD groups in ON and OFF conditions, a Two-way Mixed ANOVA was conducted in Python. For the healthy group, data imputation from the OFF to ON condition was performed, as the measurement of features was conducted only once. Differences in OFF and ON conditions within the PD group were assessed using a paired T-test for dependent samples in MS Excel 2019. The comparison between the healthy group and both conditions was conducted using an independent T-test in MS Excel 2019. Comparison of data obtained from the sEMG sensor of each forearm and each muscle group was performed separately in the PD and healthy groups. In all data, a Bonferroni-adjusted p value of <0.0125 was statistically significant. Spearman's correlation analysis was also performed in MS Excel 2019 to examine the relationship between MDS-UPDRS III scores and the number of peaks and the average time in OFF and ON conditions.

## 3 Results

### 3.1 Developed metrics

The results of Two-Way Mixed ANOVA for developed features in both groups are presented in Table 2. The effect of levodopa intake and group affiliation on the increase in the number of peaks is statistically significant for all muscle groups in the "finger tapping" and "hand movements" tests.

**Table 2:** Results of Two-Way Mixed ANOVA test.

Muscle group		Source	Number of peaks				Average time				Bonferroni-Adjusted Alpha
			DF1	DF2	F	p-value	DF1	DF2	F	p-value	
**"Finger tapping"**	**Flexors Left**	Group	1	11	18.61	0.0012	1	11	10.84	0.0072	0.0125
		Condition	1	11	23.97	0.0005	1	11	8.26	0.0151	0.0125
		Interaction	1	11	27.96	0.0003	1	11	9.63	0.0100	0.0125
	**Extensors Left**	Group	1	11	13.91	0.0033	1	11	8.36	0.0147	0.0125
		condition	1	11	19.23	0.0011	1	11	6.37	0.0283	0.0125
		Interaction	1	11	22.43	0.0006	1	11	7.43	0.0197	0.0125
	**Flexors Right**	Group	1	11	9.03	0.0120	1	11	4.95	0.0479	0.0125
		condition	1	11	64.98	0.00001	1	11	3.52	0.0874	0.0125
		Interaction	1	11	75.82	0.000003	1	11	4.11	0.0676	0.0125
	**Extensors Right**	Group	1	11	12.24	0.0050	1	11	5.96	0.0327	0.0125
		condition	1	11	64.98	0.00001	1	11	4.22	0.0644	0.0125
		Interaction	1	11	75.82	0.000003	1	11	4.93	0.0484	0.0125
**"Hand movements"**	**Flexors Left**	Group	1	11	12.41	0.0048	1	11	8.46	0.0142	0.0125
		condition	1	11	18.33	0.0013	1	11	3.73	0.0797	0.0125
		Interaction	1	11	21.38	0.0007	1	11	4.35	0.0611	0.0125
	**Extensors Left**	Group	1	11	13.24	0.0039	1	11	10.19	0.0086	0.0125
		condition	1	11	12.04	0.0052	1	11	3.30	0.0965	0.0125
		Interaction	1	11	14.04	0.0032	1	11	3.85	0.0754	0.0125
	**Flexors Right**	Group	1	11	11.94	0.0054	1	11	13.39	0.0038	0.0125
		condition	1	11	14.16	0.0031	1	11	16.67	0.0018	0.0125
		Interaction	1	11	16.53	0.0019	1	11	19.45	0.0010	0.0125
	**Extensors Right**	Group	1	11	17.58	0.0015	1	11	12.15	0.0051	0.0125
		condition	1	11	11.67	0.0058	1	11	8.20	0.0154	0.0125
		Interaction	1	11	13.62	0.0036	1	11	9.57	0.0102	0.0125

The influence of levodopa and the disease on the decrease in average time was significant only for the right forearm flexors group (Flexors R) in the "hand movements" test. For other muscle groups in the tests, the influence was not confirmed by the p-value. The change in median amplitude before and after levodopa intake in the superficial muscles of the anterior forearm (Flexors) and posterior forearm (Extensors) did not reach the level of statistical significance on both arms.

**Fig. 5:**
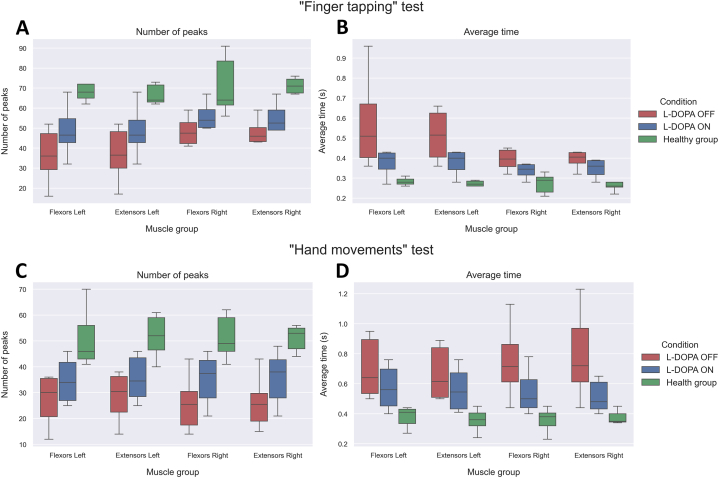
Boxplots of the resulting metrics in all groups of participants. A) Number of peaks corresponding to the moments of muscle activation in the "finger tapping" test. B) Average time interval between found peaks in the "finger tapping" test. C) Number of peaks corresponding to the moments of muscle activation in the "hand movements" test. D) Average time interval between found peaks in the "hand movements" test.

The results of post-hoc tests are presented in Table 3. For each muscle group of each test the increase in the number of peaks in the ON condition compared to OFF was statistically significant. This may indicate an increase in velocity of voluntary movements after the administration of levodopa. At the same time, the difference between the OFF condition and the healthy group was also statistically significant in each condition. It is worth noting that there is no statistically significant difference between the ON condition and the healthy group. Overall, the number of peaks after taking levodopa approaches the indicators of the healthy group (Fig. 5 A for "Finger tapping", Fig. 5C for "Hand movements").

**Table 3:** Results of post-hoc tests.

Number of peaks					
Test	Muscle group	p-value OFF and healthy	p-value ON and healthy	p-value OFF and ON	Bonferroni-Adjusted Alpha
**"Finger tapping"**	Flexors Left	0.002	0.015	0.0046	0.0125
	Extensors Left	0.002	0.024	0.0073	0.0125
	Flexors Right	0.006	0.026	0.0005	0.0125
	Extensors Right	0.003	0.015	0.0005	0.0125
**"Hand movements"**	Flexors Left	0.002	0.014	0.0080	0.0125
	Extensors Left	0.001	0.010	0.0180	0.0125
	Flexors Right	0.002	0.016	0.0135	0.0125
	Extensors Right	0.001	0.010	0.1900	0.0125
**Average Time**					
**Test**	**Muscle group**	**p-value OFF and healthy**	**p-value ON and healthy**	**p-value OFF and ON**	**Bonferroni-Adjusted Alpha**

**"Finger tapping"**	Flexors Left	0.028	0.039	0.036	0.0125
	Extensors Left	0.042	0.042	0.054	0.0125
	Flexors Right	0.095	0.074	0.122	0.0125
	Extensors Right	0.072	0.061	0.097	0.0125
**"Hand movements"**	Flexors Left	0.053	0.024	0.113	0.0125
	Extensors Left	0.041	0.018	0.131	0.0125
	Flexors Right	0.010	0.027	0.010	0.0125
	Extensors Right	0.016	0.058	0.036	0.0125

The reduction in average time in the ON condition compared to OFF was not statistically significant. The same results are observed in the comparison of OFF and ON with the healthy group. Despite the lack of statistical significance, there is a tendency towards a reduction in the average time in the ON condition, approaching the indicators of the healthy group (Fig. 5 B for "Finger tapping", Fig. 5 D for "Hand movements").

### 3.2 Correlation between score and developed features

To investigate the relationship between changes in MDS-UPDRS III scores and sEMG features, Spearman correlation coefficients were calculated for the difference in scores before and after drug administration. The analysis compared the difference in the number of peaks and the average time during levodopa OFF and ON conditions. Results of Spearman correlations are presented in Table 4.

**Table 4:** Results of Spearman correlations.

Correlations	Muscle groups	"Finger tapping" test				Bonferroni-Adjusted Alpha
		Number of peaks		Average time		
		Spearman correlation	p-value	Spearman correlation	p-value	
**OFF (total scores)**	Flexors Left	−0.84	0.036	0.77	0.0724	0.0125
	Extensors Left	−0.81	0.0499	0.77	0.0724	0.0125
	Flexors Right	−0.89	0.019	0.89	0.0188	0.0125
	Extensors Right	−0.89	0.019	0.89	0.0188	0.0125
**ON (total scores)**	Flexors Left	−0.77	0.072	0.77	0.0724	0.0125
	Extensors Left	−0.77	0.072	0.77	0.0724	0.0125
	Flexors Right	−0.94	0.005	0.94	0.0048	0.0125
	Extensors Right	−0.93	0.008	0.94	0.0048	0.0125
**OFF (scores for upper limbs items)**	Flexors Left	−0.66	0.152	0.58	0.2278	0.0125
	Extensors Left	−0.62	0.191	0.87	0.0244	0.0125
	Flexors Right	−0.94	0.005	0.94	0.0051	0.0125
	Extensors Right	−0.94	0.005	0.94	0.0051	0.0125
**ON (scores for upper limbs items)**	Flexors Left	−0.88	0.020	0.53	0.2798	0.0125
	Extensors Left	−0.88	0.020	0.88	0.0198	0.0125
	Flexors Right	−0.84	0.036	0.84	0.0361	0.0125
	Extensors Right	−0.90	0.015	0.84	0.0361	0.0125

In the "hand movements" test, a negative correlation was observed between the MDS-UPDRS III scores and the number of peaks in the ON condition. In the right arm, negative correlations were found for both flexors and extensors, reaching statistical significance (Z = −0.928, p = 0.008). However, in the left arm of both muscle groups, the correlations did not reach the level of significance (Z = −0.5 p > 0.05 for Flexors and Z = −0.43 p > 0.05 for Extensors). As for the "finger tapping" test, no correlation was found between the changes in scores and the difference in the number of peaks.

The correlation between the differences in average time (in ON and OFF conditions) and the difference in the scale scores did not reach statistical significance in both tests and on both arms.

The relationship between the developed features and the total scores of MDS-UPDRS III and scores for upper limb tests in both OFF and ON conditions, as well as for each side, was explored. The scores for upper limb tests were composed of MDS-UPDRS III items 3.3b, 3.3c ("rigidity" - right and left extremity), 3.4 ("finger tapping"), 3.5 ("hand movements"), 3.6 ("pronation-supination movements") 3.15 ("postural tremor"), 3.16 ("kinetic tremor"), 3.17a, and 3.17b ("rest tremor amplitude" - right and left extremity).

**Fig. 6:**
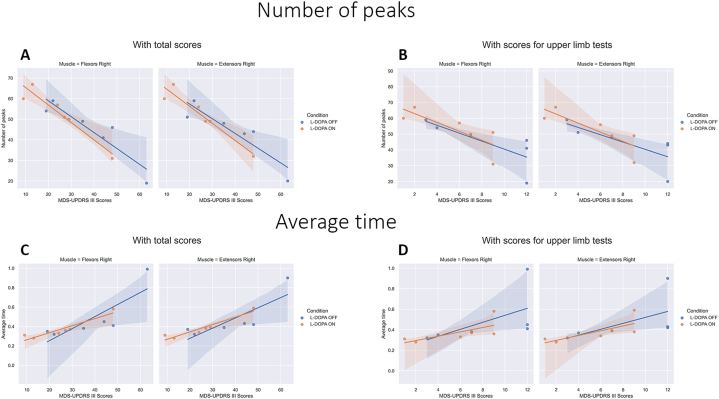
Correlation plots for the "finger tapping" test. A) Correlation between the difference in number of peaks (in both conditions) and the difference in the total scores of MDS-UPDRS III. B) Correlation between the difference in number of peaks (in both conditions) and the difference in the scores for upper limb test of MDS-UPDRS III. C) Correlation between the difference in average time (in both conditions) and the difference in the total scores of MDS-UPDRS III. D) Correlation between the difference in average time (in both conditions) and the difference in the scores for upper limb test of MDS-UPDRS III.

During all combinations, statistical significance was observed in the flexor and extensor muscles of the right forearm. Notably, a negative correlation was observed in the OFF condition when analyzing the number of peaks with the total MDS-UPDRS III scores (Z = −0.94; p = 0.005 for Flexors and Z = −0.93; p = 0.008 for Extensors) (Fig. 6 A), and in the ON condition when analyzing scores for upper limb tests (Z = −0.94; p = 0.005 for Flexors and Extensors) (Fig. 6 B). In other cases, the correlation coefficient was near the level of statistical significance. It is worth mentioning that such results were observed only in the "finger tapping" test.

In all conducted combinations, a positive correlation coefficient was observed for the average time. In the "hand movements" test, statistical significance was not achieved. In the "finger tapping" test, a high correlation coefficient was identified on the right forearm with the total scores in the OFF condition (Z = −0.94; p = 0.005 for Flexors and Extensors) (Fig. 6C) and with scores for upper limb tests in the ON condition (Z = −0.94; p = 0.005 for Flexors and Extensors) (Fig. 6 D). In other cases, the correlation coefficient approached the threshold of statistical significance.

### 3.3 Model

**Fig. 7:**
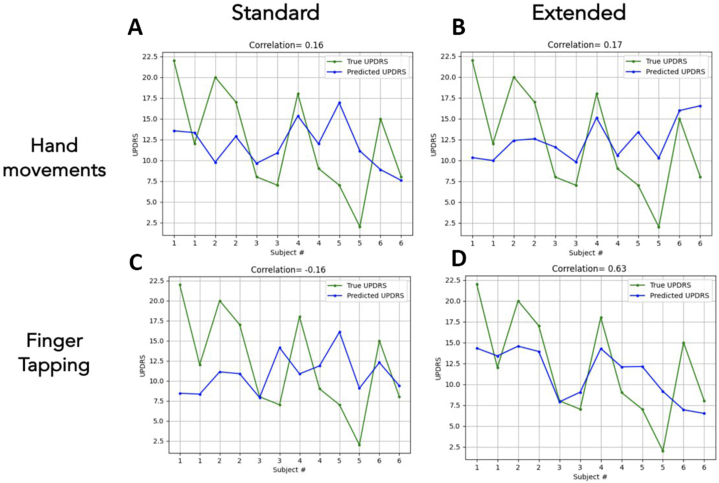
Plots of model score prediction. A) Training on a standard set ("hand movements" test). B) Training on an extended set ("hand movements" test). C) Training on a standard set ("finger tapping" test). D) Training on an extended set ("finger tapping" test).

With the standard metrics RF model predicted MDS-UPDRS III score with the correlation of 0.16 for the "hand movements" test (Fig. 7 A) and with the correlation of −0.16 for "finger tapping" test (Fig. 7C). When additional metrics were included in the extended model, the correlation reached to 0.17 for the "hand movements" test (Fig. 7 B) and with the correlation reached to 0.63 for "finger tapping" test (Fig. 7 D).

## 4 Discussion

PD is characterized by a motor symptom known as bradykinesia, which manifests as a generalized decrease in the velocity of voluntary movements. Bradykinesia in PD patients is associated with dysfunction of the basal ganglia, namely a significant (about 50–60 %) depletion of dopamine in the striatum [29]. This results in dysfunction of neuronal networks affecting the basal ganglia, cortical areas, and the cerebellum [29]. In our study, we utilized the sEMG method to identify patterns indicative of changes in motor function during "finger tapping" and "hand movements" tests in PD patients on medication-assisted levodopa OFF and ON conditions. Data from sEMG sensors could potentially offer an objective quantitative method for evaluating muscle function. The application of sEMG in the clinical settings will enable the determination of differences in motor function following surgical and therapeutic interventions, as well as the assessment of rehabilitation effectiveness. The method's advantages include noninvasiveness and mobility. Recent advancements in wearable sensor technologies allow the recording of sEMG data outside the laboratory, capturing natural conditions during daily activities [30].

In studies focused on motor symptoms in PD, sEMG is commonly employed to detect tremor and less frequently for assessing bradykinesia [21]. However, in addition to the tremor-dominant subtype of PD, where tremor predominates over other motor symptoms, there is also an akinetic-rigid subtype, characterized by weakly expressed tremor and noticeable bradykinesia, as well as a mixed subtype. Evidence suggests the prevalence of the akinetic-rigid form in patients from the middle of the sixth decade [31]. Therefore, an objective quantitative assessment method should combine the assessment of tremor and bradykinesia.

IMU data are mostly used to assess bradykinesia [32,33]. However, the need to place IMU sensors on the fingers and hand during active "hand movements" tests can create additional stress and affect the result, whereas sEMG sensors are placed on the forearm. Therefore, we decided to use sEMG to assess bradykinesia.

We have developed metrics that provide an objective assessment of the severity of bradykinesia. We observed an increase in the number of peaks (maxima), indicating elevated muscle activation, after levodopa intake. Additionally, a decrease in the average time between peaks in the ON condition was observed. These features suggest a reduction in bradykinesia following levodopa intake and may serve as a diagnostic criterion for quantifying this motor symptom. When analyzing the average time muscle activations, it is not only the average duration of the interval that matters, but also the duration of each interval in comparison to others. This approach enables the detection of movement delays and interruptions in sEMG signals, potentially allowing for a direct correlation with MDS-UPDRS III scores.

Correlations between changes in the number of peaks and MDS-UPDRS III scores in the levodopa ON and OFF conditions are obtained even on a small sample. This indicates that the MDS-UPDRS III scores and our proposed parameters are comparable.

We validated the contribution of the features we developed for model training. We were able to reproduce the results from the original study [21] on our data. The use of the extended set showed good results on the "finger tapping" test. In this test, the model trained on the standard set did not predict scores with sufficient correlation. This may be related to the fact that we conducted this test differently. In the authors of the original study, subjects tapped their index finger on a tablet placed on a table with their hand fixed on the wrist [21]. In our study the subjects' hand was balanced in the air. This, combined with mechanical artifacts due to intensive fluctuations of the subcutaneous fatty tissue, introduced additional interference into the signal. Nevertheless, we consider our approach to the test to be reasonable since it is similar to the clinical test and allows no additional testing to be performed during the clinical assessment according to MDS-UPDRS III. However, the model trained on the extended set demonstrated high reliability in predictions.

In the "hand movements" test, the model did not demonstrate high accuracy in predicting MDS-UPDRS III scores, which is an expected outcome. On the one hand, our features did not show a statistically significant correlation with MDS-UPDRS III scores. On the other hand, the authors of the original study used a set of metrics exclusively for assessing the "finger tapping" test [21], and there is a possibility that these features are not suitable for evaluating the "hand movements" test.

The integration of many features to assess the regularity and rhythmicity of the sEMG signal, as well as the metrics we developed, increases the efficiency of the predictions in both tests. Such results may indicate the prospect of the new metrics in further use for training various models that automate the scoring of MDS-UPDRS III.

In the current study, we present the results based on a group of six patients. For a more detailed comprehensive evaluation of the proposed methods in the subsequent phase, it is imperative to expand the participant sample. Furthermore, the inclusion of a control group of PD patients who are not taking levodopa during the experiment is necessary. Additionally, assessments of motor symptoms according to MDS-UPDRS III were conducted by a single neurologist, introducing a degree of subjectivity. To obtain a more complete and objective understanding of impairments, assessments from multiple experienced neurologists are necessary.

It's also worth noting that in this study, we assessed bradykinesia in isolation, defining it as a reduction in the speed of voluntary movements. However, parkinsonism is typically characterized by the combination of bradykinesia with manifestations such as the sequence effect or hypokinesia, whereas isolated bradykinesia is common across a broad spectrum of conditions unrelated to parkinsonism. In order for the proposed metric to be suitable for differential diagnosis, additional evaluation of hypokinesia and the sequence effect is necessary, which could be the subject of further research.

## 5 Conclusion

In summary, this study observed a reduction in bradykinesia during the "finger tapping" and "hand movements" tests of MDS-UPDRS III after the administration of levodopa in patients with PD. This reduction was characterized by an increase in the number of peaks in the sEMG signal. This feature partially correlates with MDS-UPDRS III scores and enhances the similarity of predictions made by model with clinician assessments in the "finger tapping" test. Our results demonstrate the potential of using sEMG for assessing bradykinesia and the promise of this method in enhancing the objectivity of MDS-UPDRS III.

## Data availability

All data and scripts to this article can be found online at https://github.com/MorganStingray/Kozulin-sEMG_PD.

## Funding sources for study

This work was supported by Far Eastern Federal University Endowment Fund dated 05.24.2022 №D-166-22; Project №23-044-2.06-0008 "Scientific, educational and testing ground: artificial intelligence and digital medical services (FEFU AI Testing Ground: MedTech)" as part of the "Digital Development Center" of the strategic academic leadership program "Priority 2030"; the Russian Science Foundation under grant № 21-75-30024.

## CRediT authorship contribution statement

**Nikita Kozulin:** Writing – original draft, Visualization, Validation, Formal analysis, Data curation, Conceptualization. **Anastasiya Migulina:** Writing – original draft, Visualization, Validation, Formal analysis, Data curation, Conceptualization. **Denis Mokrushin:** Validation, Formal analysis, Data curation. **Gurgen Soghoyan:** Writing – review & editing, Writing – original draft, Visualization, Validation, Methodology, Formal analysis, Data curation, Conceptualization. **Aleksandr Artemenko:** Methodology, Formal analysis. **Artur Biktimirov:** Writing – review & editing, Supervision, Project administration, Funding acquisition, Conceptualization.

## Declaration of competing interest

The authors declare the following financial interests/personal relationships which may be considered as potential competing interests: Artur Biktimirov reports financial support was provided by 10.13039/501100012190Ministry of Science and Higher Education of the Russian Federation. Gurgen Soghoyan reports financial support was provided by 10.13039/501100006769Russian Science Foundation. If there are other authors, they declare that they have no known competing financial interests or personal relationships that could have appeared to influence the work reported in this paper.
