# Supplementary material for: Identification of electromyographic patterns of bradykinesia in patients with Parkinson's disease
Source: Heliyon. 2024 Oct 5;10(20):e39014. doi: 10.1016/j.heliyon.2024.e39014 (PMC11620153; doi:10.1016/j.heliyon.2024.e39014)
Supplement: Multimedia component 1 [file mmc1.docx]

**Inclusion and Exclusion criteria**

| Inclusion criteria | Exclusion criteria |
| --- | --- |
| Confirmed diagnosis of PD  Signed informed consent obtained  Abstinence from levodopa for at least 12 hours | Patient's refusal to participate in the study  Presence of other neurological disorders with similar clinical manifestations  Drug addiction (including in the medical history)  Congenital upper limb deformities  Central nervous system developmental anomalies  Presence of psychiatric disorders (including in the medical history)  History of cancer  History of epilepsy |
